# Supplementary material for: Dynamics of pH at Santa Catalina Island
Source: PLoS One. 2023 Dec 7;18(12):e0290039. doi: 10.1371/journal.pone.0290039 (PMC10703214; doi:10.1371/journal.pone.0290039)
Supplement: S1 File — (PDF) [file pone.0290039.s001.pdf]

Supplementary materials for “Dynamics of pH at Santa Catalina Island”

1. Calibration

Analysis of calibration results indicates a bias in the pH measurements. The sensors were calibrated 47 times during the deployments that provided data for the “Dynamics of pH at Santa Catalina Island” study. Every time the sensors were calibrated, a pre-calibration value (that is, the measurement of the calibration standard before adjustment for the present calibration) was recorded. The differences between the standard and pre-calibration values were analyzed and plotted as a function of the time between calibrations, shown in Figure S-1. The differences do not appear to have a temporal dependence. The statistics of the differences are shown in Figure S-2. The mean and standard deviation of the changes were 0.040 and 0.063, respectively for a pH 7 buffer and 0.037 and 0.065, respectively for the pH 10 buffer. The differences at the two pH values are highly correlated as seen in Figure S-3. The interpolated bias for seawater pH (8.15) is 0.039. The mean values are considered a bias to be adjusted for in analysis of mean pH values at the island, which is implemented as a subtraction of 0.04 from the measured value.

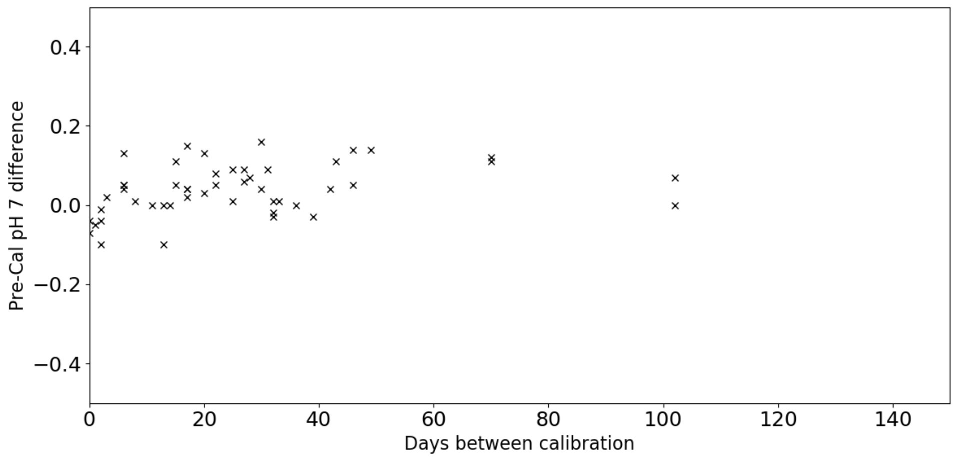

Figure S-1. Difference between Standard Buffer measurement and Buffer values for pH = 7 versus days between calibrations.

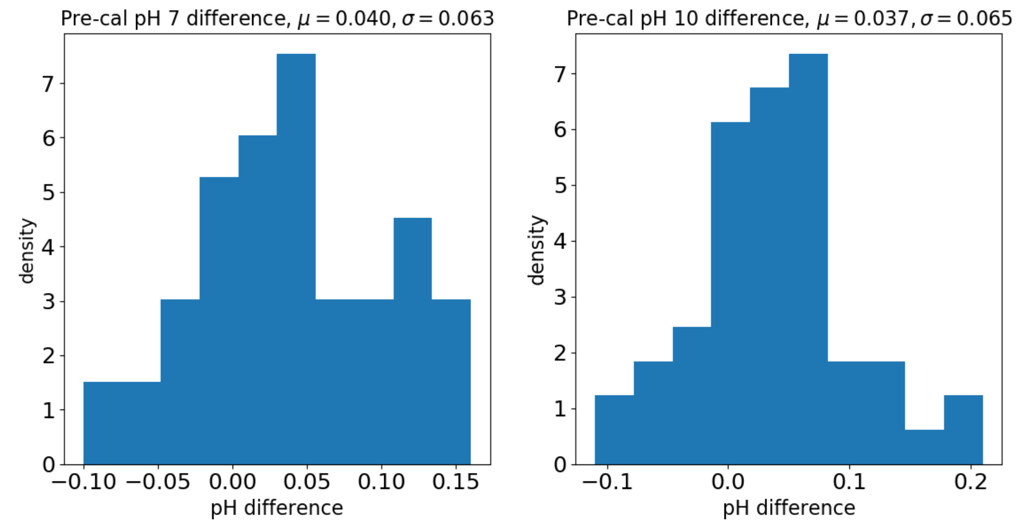

Figure S-2. Statistics of (Pre-calibration – standards) values for pH buffer 7 (left) and 10 (right).

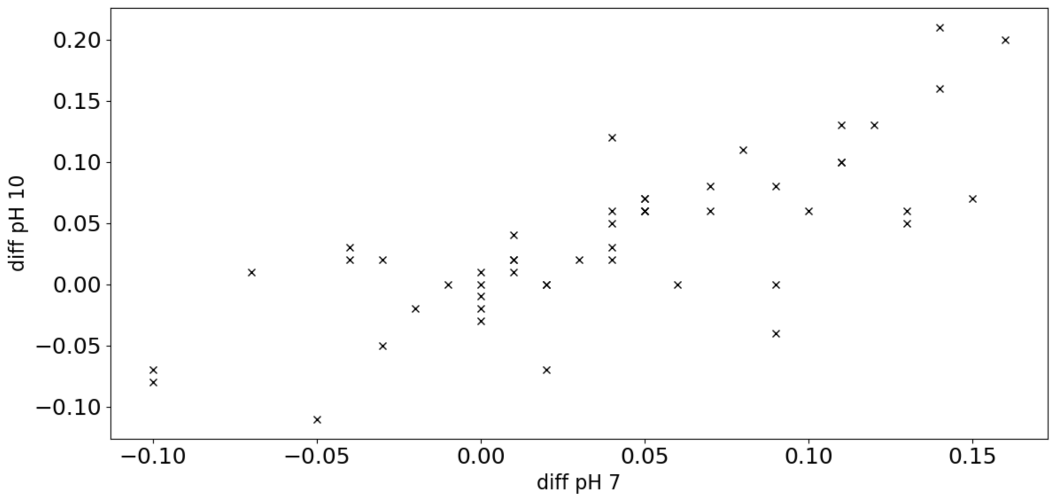

Figure S-3. Pre-calibration – standards values for buffers pH 7 vs pH 10.

2. CalCOFI salinity depth gradient

Examples of salinity profiles near Santa Catalina indicating upward gradients.

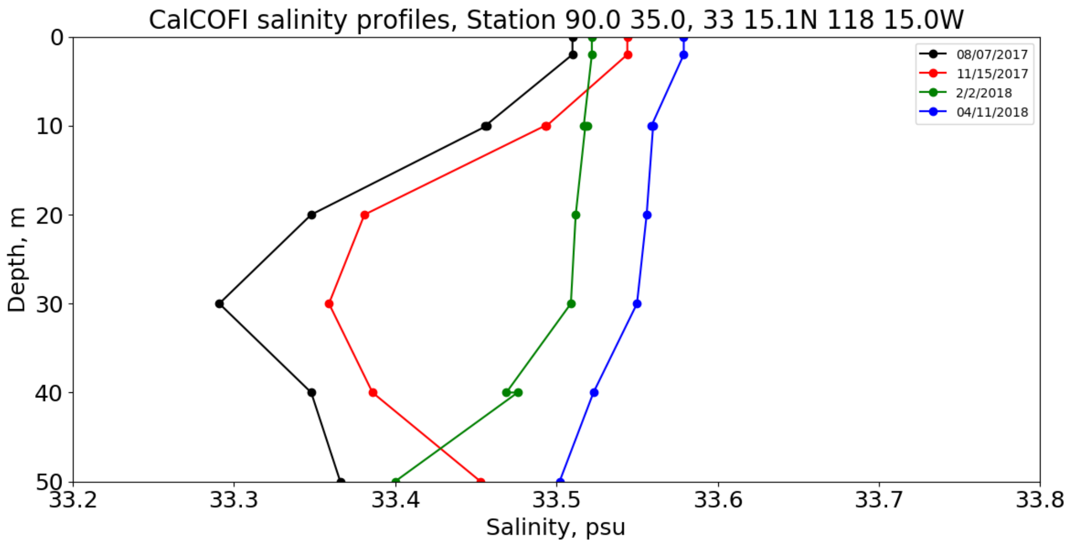

Figure S-4. CalCOFI salinity profiles.
