# Supplementary material for: Dynamics of pH at Santa Catalina Island
Source: PLoS One. 2023 Dec 7;18(12):e0290039. doi: 10.1371/journal.pone.0290039 (PMC10703214; doi:10.1371/journal.pone.0290039)
Supplement: S2 File — (PDF) [file pone.0290039.s002.pdf]

CMS YSI EXO Calibration Procedure

September 2018

Table of Contents

1. Introduction ……………………………………………… 2

2. Preparation……………………………………………….. 4

3. Calibration Setup……………………………………… 5

4. Conductivity Calibration………………………………… 7

5. pH Calibration…………………………………………… 11

6. Dissolved Oxygen Calibration…………………………... 15

7. Depth setting……………………………………………. 17

8. Chlorophyll Calibration ………………………………… 17

9. Recording Results……………………………………….. 17

10. Deployment Settings……………………………………. 18

11. Maintenance…………………………………………….. 22

1. Introduction

Thank you for participating in our field programs. Calibrating and recording the calibration data are essential to producing good research. Understanding the calibration results enables us to interpret the field measurements, understand the health of our instruments, and justify our research conclusions. Our work would not be possible if not for the efforts of volunteers like you.

This manual is written specifically for the YSI EXO and the Catalina Marine Society's use of it for its Catalina Dynamic Ocean Chemistry program. We have organized the procedure so that the work is straightforward and efficient.

However, please **have an experienced calibrator go through the procedure with you**. There are always tricks and techniques that are best shown rather than written.

2. Preparation

The calibration and deployment procedures will be easier if the workspace is organized; non-needed materials removed and needed materials laid out in an easily accessible arrangement. The preparation for a typical CMS depth-profiling deployment includes knowledge of where, when and how it is to be deployed. Below is a checklist of the items needed for a typical calibration.

☐ Type of deployment: depth-profiling or mooring required to set sampling interval

☐ Where: Avalon, Two Harbors, etc for internal documentation

☐ Date and time for deployment

☐ Access to local atmospheric pressure readings

☐ Computer with KOR software and EXO USB adapter or Bluetooth

☐ Cleaning clothes

☐ Sharpie or permanent marker

☐ Pen and paper

☐ Tools (brush, battery wrench, and sensor-removal tool, Figure 2-1)

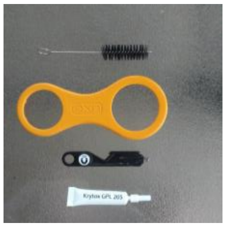

Figure 2-1. Brush, battery tool, sensor tool and grease

☐ 4 D batteries

☐ 50,000 μSiemens/cm solution for rinsing

☐ 50,000 μSiemens/cm solution for calibration measurement

☐ pH 7.00 buffer for rinsing

☐ pH 7.00 buffer for calibration measurement

☐ pH 10.00 buffer for rinsing

☐ pH 10.00 buffer for calibration measurement

☐ Krytox grease

Nice to have:

EXO User's Manual

Internet access

3. Calibration Setup

3.1 Replace the batteries in the sonde

a) Remove the battery cap at the end of the sonde using the EXO tool, dump the batteries and replace with 4 fresh D batteries. Do not drop batteries vertically into the tube; but slant the tube so the batteries fall slowly and do not damage the spring at the bottom of the battery tube.

b) Inspect the battery O rings, remove with a credit card, clean the threads, grease the O rings and replace the O rings.

c) Replace battery cap and tighten with tool.

3.2 Check sensor connections

a) Remove the calibration cup and the cage guard. Check the sensors for caps and remove and set aside the caps and covers if any are found. (need pictures)

b) Test the connections between the sensors and the sonde with your fingers. If loose, tighten with the sensor-removal tool or pliers.

3.3 Clean the sensors

a) Remove any debris from the sensors; check the pH, Optical Dissolved Oxygen (ODO), and the hole in the temperature/conductivity sensor. Clean the hole with the brush.

3.4 Attach EXO USB Adapter and execute KOR application

a) Remove rubber plug on right side of sonde and plug in the EXO USB Adapter.

b) Attach USB cable to adapter and also into computer. (Figure 3-1)

c) Open the KOR application. It should identify the COMM field that communicates with the sonde. Select it and click “Connect”

d) Then click the “Run”   and then “Dashboard” to see the measurements being processed by the sonde. You are now interfacing with the sonde.

If using Bluetooth, use the magnet on the sensor-removal tool (see picture) to turn the sonde’s Bluetooth on. Connect and see the dashboard (Figure 3-2).

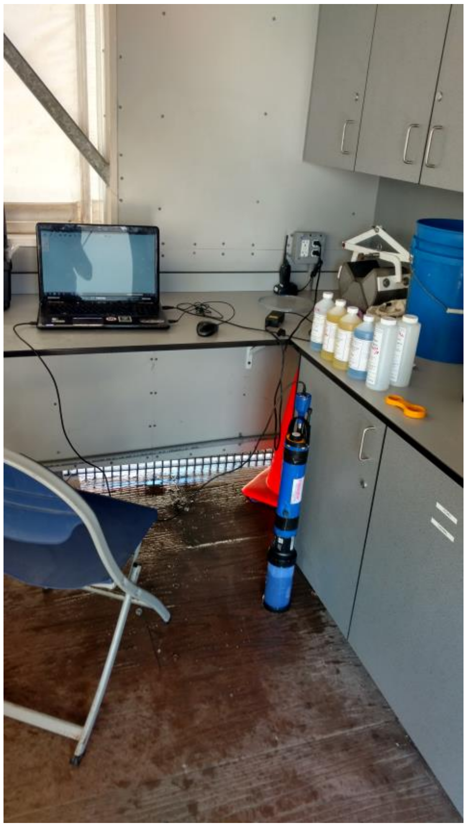

Figure 3-1. Laptop connected to sonde via cable interface

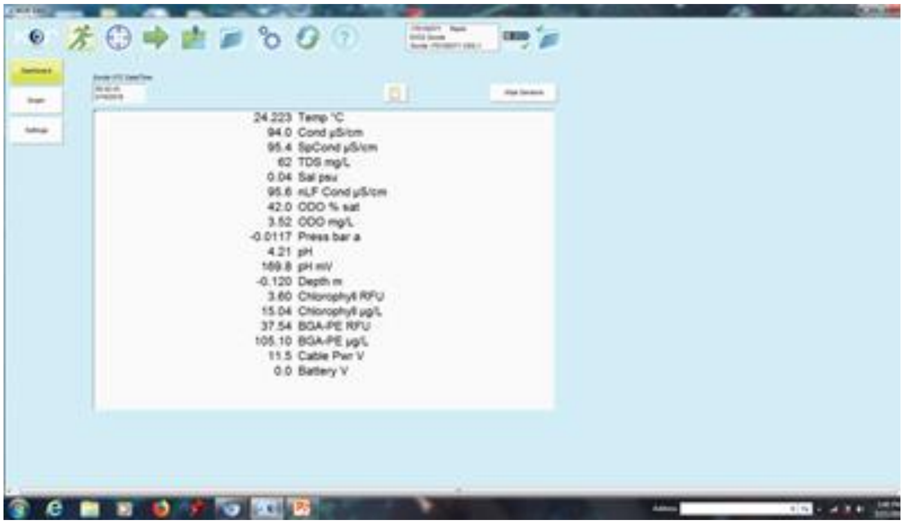

Figure 3-2. Screen indicating successful interface to sonde.

4. Conductivity Calibration

Conductivity calibration is performed by measuring a solution of known conductivity and then adjusting the sonde to yield the true value. The sensor must be cleaned of all contaminating fluids that could change the conductivity of the calibration solution. The cal solution is typically 50,000 µSiemens/cm (microSiemens/cm). This value of specific conductivity is close to that of sea water.

4.1 Rinsing Sonde with cal solution

The purpose of rinsing the sonde is to remove or dilute all fluid in the sonde that may affect calibration. First, leaving the guard cage on the sonde, remove the calibration cup and fill it with 1/3 of the 50000 µSiemens/cm rinse solution. This could be the recaptured calibration solution used in previous calibrations. Place the sonde in the cup and screw the collar tight. Then shake the sonde, rinsing all sensors and crevices on the sensor head (shake 15 seconds or so). Discard the rinse solution, fill the cal cup again, and rinse. The sensors should be rinsed 3 times.

Next, open the fresh solution and mark the date on the container and write an "R" on the cap (Figure 4-1). Fill the cal cup with fresh solution using the contents of the entire container. Put the sonde in the cal cup, making sure the temperature/conductivity sensor is covered in solution.

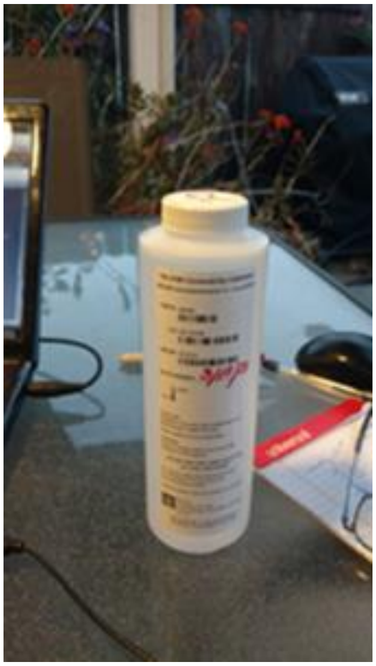

Figure 4-1. Conductivity solution marked after opening.

4.2 Calibration Procedure

In the KOR application, click the “calibration” icon. The list of ports with sensors will appear as shown in Figure 4-2. Click “Conductivity” which will bring up another screen describing the calibrations options for that sensor. Click “SpCond µS/cm”.

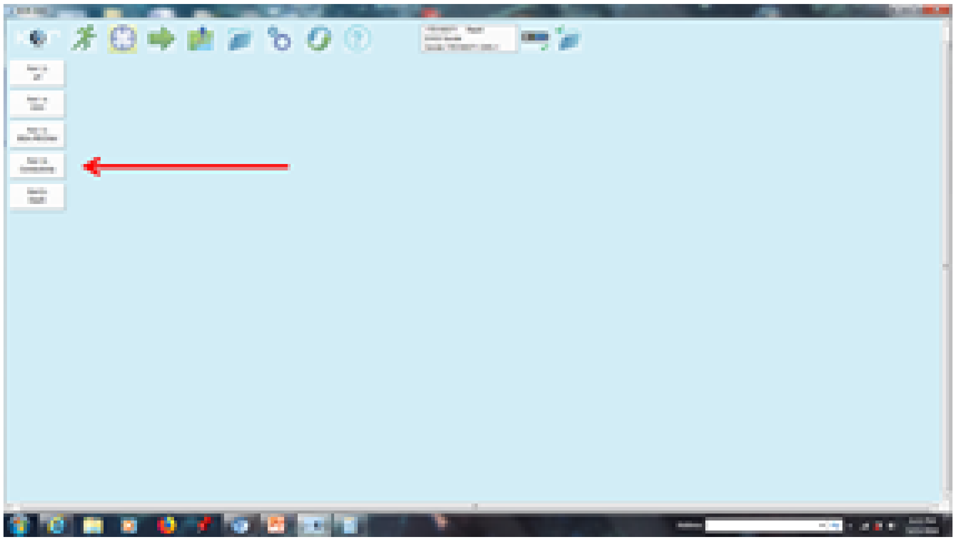

Figure 4-2. use SpCond for calibration.

A screen for data input will appear as in Figure 4-3

Click 1 Point Calibration and fill in the column with the solution specific conductivity (e.g., 50000), lot number, chemical ingredient and manufacturer. Then click “Start Cal”

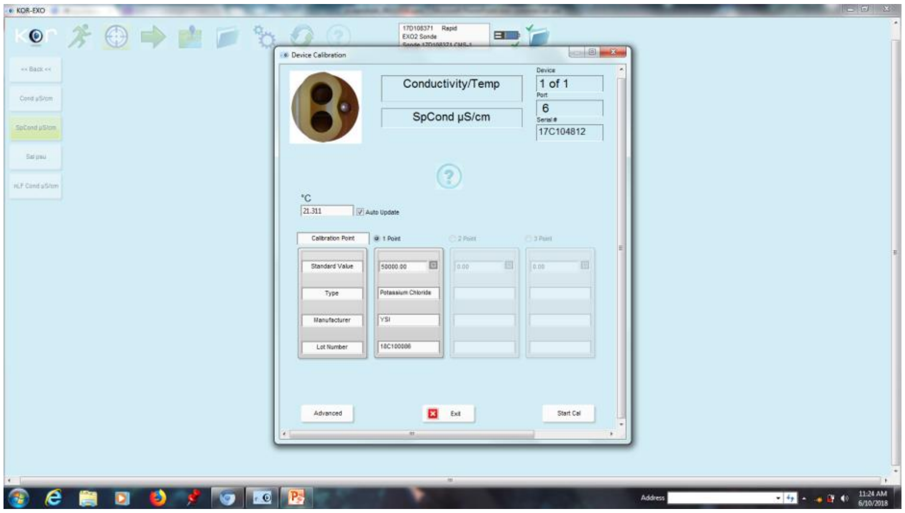

Figure 4-3. Input screen for conductivity calibration

The following screen (Figure 4-4) should appear. It will indicate Unstable data until the readings stabilize. When they stabilize, click "Apply".

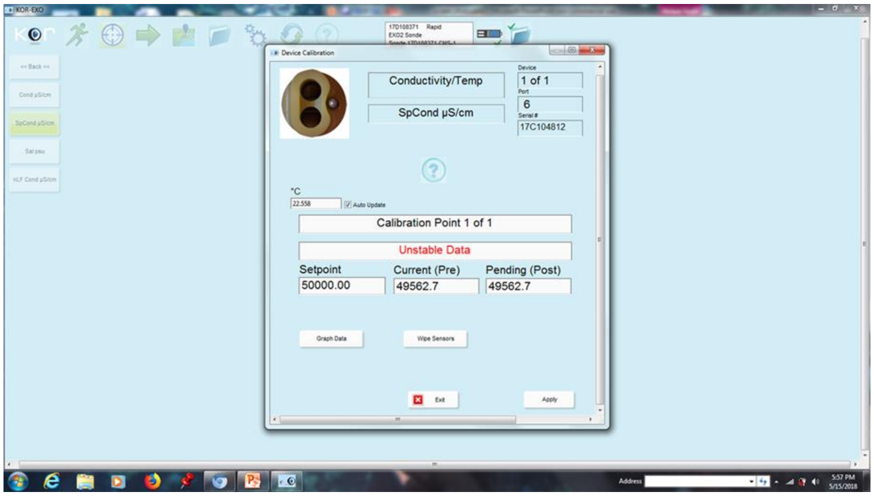

Figure 4-4.

Next, click “View Worksheet”. If the system meets certain characteristics a green check (√) will appear on the next screen.

Print the worksheet, if you have access to a printer. Else make an electronic file and e-mail the file to the chief scientist. Then click “Exit”. The conductivity calibration is almost complete. It will be complete when the worksheet is placed on the website. If an X appears, still print the worksheet to be placed on the website. However, check the conductivity sensor and make sure it is attached securely to the sonde. If not, attach it securely and run the calibration again.

5. pH Calibration

The calibration of the pH sensor is similar to that of the conductivity sensor (Section 4), but with two important differences. First, the calibration is a 2-point procedure; and second, the pH cal solution is temperature dependent.

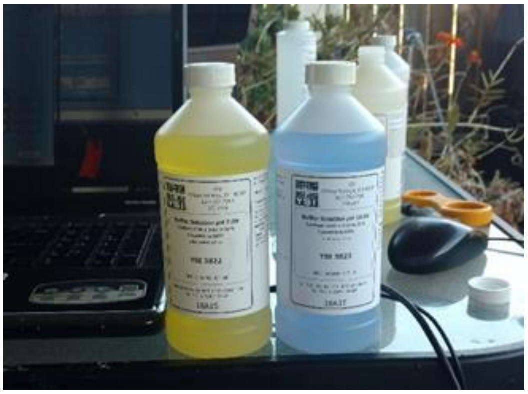

Figure 5-1. pH buffers

We use two calibration solutions (see Figure 5-1), a pH 7 buffer (dyed yellow) and a pH 10 buffer (dyed blue).

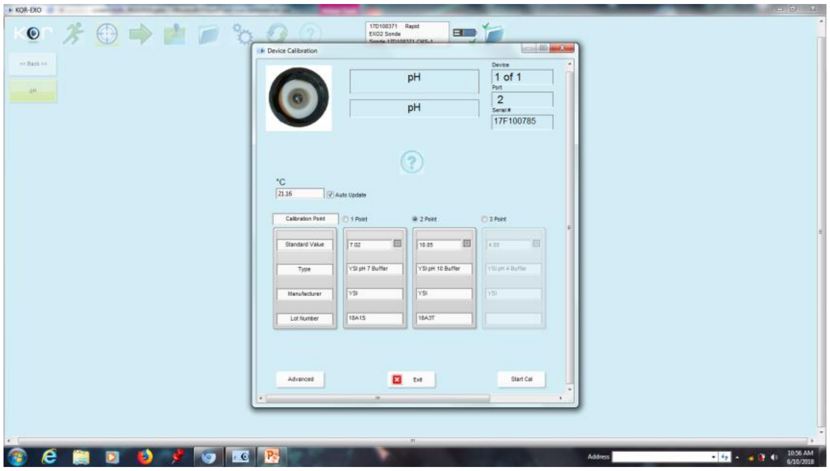

Figure 5-2. pH cal input screen

Invoke the calibration icon, click pH sensor and then click pH. You should see the input template shown in Figure 5-2. Note the temperature at the upper left of the screen. Check the 2-point circle, then fill in the blanks for both solutions. For the pH value, look on the container label and note the pH value that corresponds nearest to the temperature. Put this value in as well as the lot numbers.

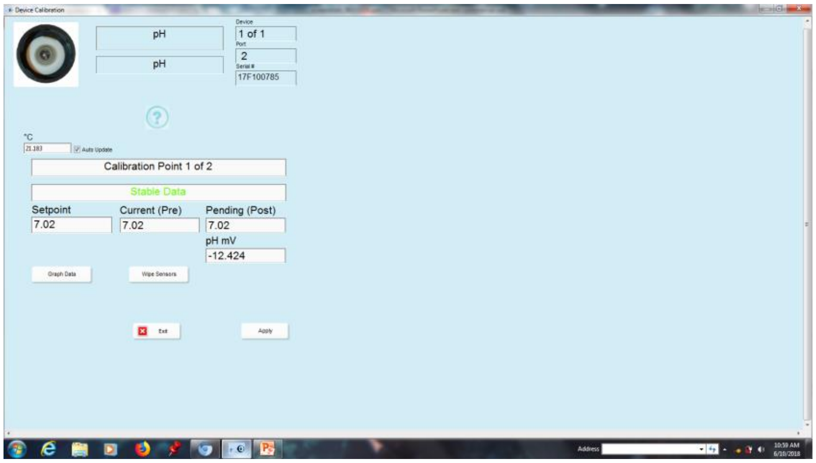

Figure 5-3.

Next, starting with pH of 7, begin the rinse procedure using the pH 7 rinse. Fill the cal cup with 1/3 of the solution, screw the cup onto the sonde, shake for 15 seconds and repeat such that the sensors are rinsed 3 times. After rinsing, fill the cup with fresh solution, place the sensors in the cup and click “Start Cal”. The screen similar to that shown in Figure 5-3 should appear, at first indicating unstable data, but after a short time will indicate stable data.

When the data readings are stable, click “apply”. Then click “Proceed” at the bottom right.

A message about proceeding to Standard 10.00 pH shows up (Figure 5-4). **Before you proceed**, rinse 3 times with the pH 10, fill calibration cup with fresh 10 pH solution, then hit “OK”.

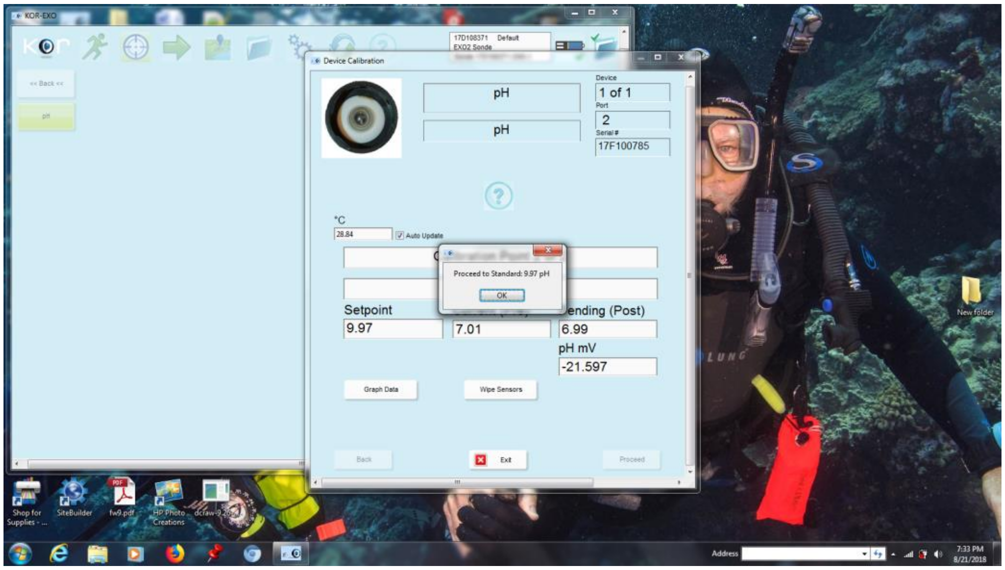

Figure 5-4. When this screen appears, begin pH 10 cal solution preparation

When ready, hit OK , then click start Cal and when data are stable (Figure 5-5), hit complete. Then view worksheet, Figure 5-6 and verify green check. If green check does not appear, checnk sensor connections. Then print and place on website (or e-mail to webmaster).

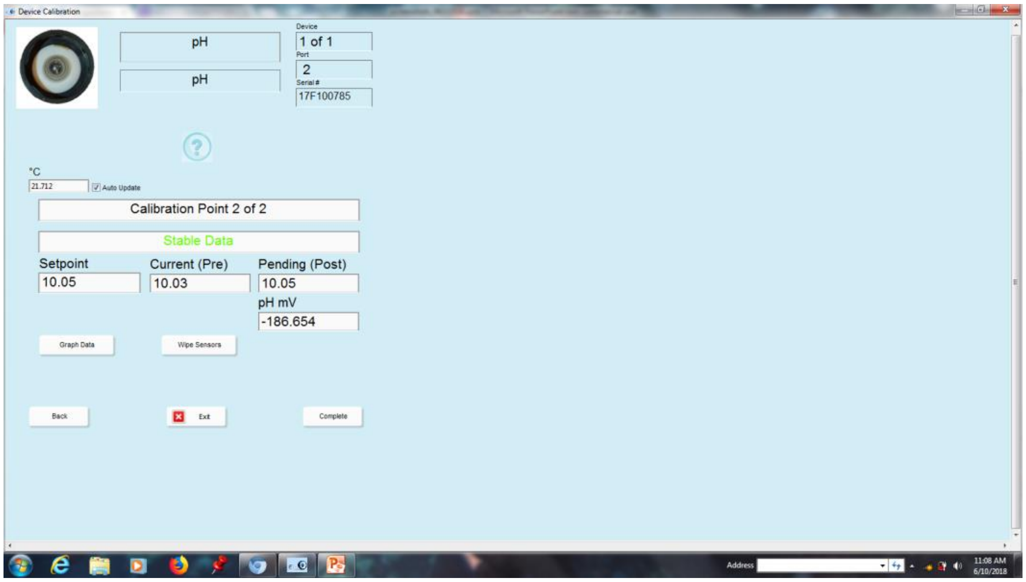

Figure 5-5. Second point pH completion

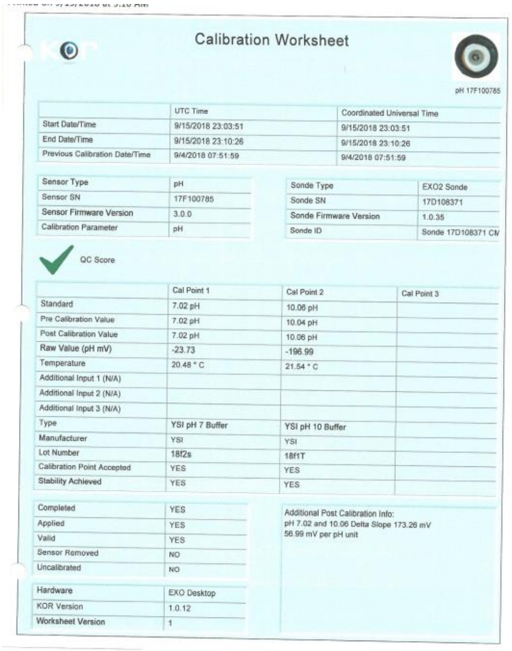

Calibration Worksheet

pH 17F100785

	UTC Time	Coordinated Universal Time
Start Date/Time	9/15/2018 23:03:51	9/15/2018 23:03:51
End Date/Time	9/15/2018 23:10:26	9/15/2018 23:10:26
Previous Calibration Date/Time	9/4/2018 07:51:59	9/4/2018 07:51:59

Sensor Type	pH
Sensor SN	17F100785
Sensor Firmware Version	3.0.0
Calibration Parameter	pH

Sonde Type	EXO2 Sonde
Sonde SN	17D108371
Sonde Firmware Version	1.0.35
Sonde ID	Sonde 17D108371 CM

QC Score

	Cal Point 1	Cal Point 2	Cal Point 3
Standard	7.02 pH	10.06 pH	
Pre Calibration Value	7.02 pH	10.04 pH	
Post Calibration Value	7.02 pH	10.06 pH	
Raw Value (pH mV)	-23.73	-196.99	
Temperature	20.48 ° C	21.54 ° C	
Additional Input 1 (N/A)			
Additional Input 2 (N/A)			
Additional Input 3 (N/A)			
Type	YSI pH 7 Buffer	YSI pH 10 Buffer	
Manufacturer	YSI	YSI	
Lot Number	18f2s	18f1T	
Calibration Point Accepted	YES	YES	
Stability Achieved	YES	YES	

Completed	YES
Applied	YES
Valid	YES
Sensor Removed	NO
Uncalibrated	NO

Hardware	EXO Desktop
KOR Version	1.0.12
Worksheet Version	1

Additional Post Calibration Info:
pH 7.02 and 10.06 Delta Slope 173.26 mV
56.99 mV per pH unit

Figure 5-6. Cal sheet for pH

6. Dissolved Oxygen Calibration

Calibration of the oxygen sensor is simple. But you need to know atmospheric pressure for your location. This can be obtained from the internet. Look for the nearest airport or weather

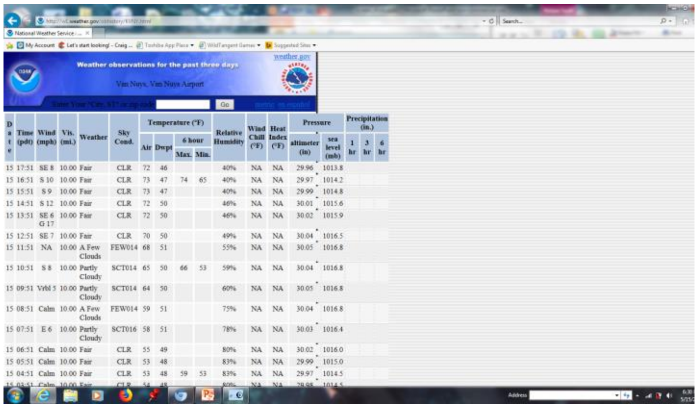

Figure 6-1. Airport weather.

station that is at your altitude. Shown in Figure 6-1 is the weather for Van Nuys airport (altitude = 802 ft) obtained from http://w1.weather.gov/obhistory/KVNY.html. Similarly for John Wayne airport (altitude = 56 ft) the pressure can be obtained from http://w1.weather.gov/obhistory/KSNA.html.

In the two columns under "Pressure", the left is the "altimeter" pressure while the right column is the "sea level pressure". Note that the altimeter pressure is in inches of mercury (inHg), while the sea level pressure is in mbar (milli bars). Both of these are the equivalent sea level pressure at the airport location. For calibration, however, we need absolute pressure at the location of calibration. Hence, we must uncorrect the reported pressure.

First, note that the KOR software wants pressure in millimeters of mercury (mmHg).

1 inch = 25.4 mm

Hence, 29.96 inHg = 760.984 mmHg. (For reference 1 mbar = 0.0295 inHg = 0.7493 mmHg) Once converted to mmHg, the pressure can be uncorrected via the following formula:

True Barometric Pressure = (Corrected Pressure) – 2.5 x local altitude in ft/100). True Barometric pressure is what is input into the KOR interface, Figure 6-2.

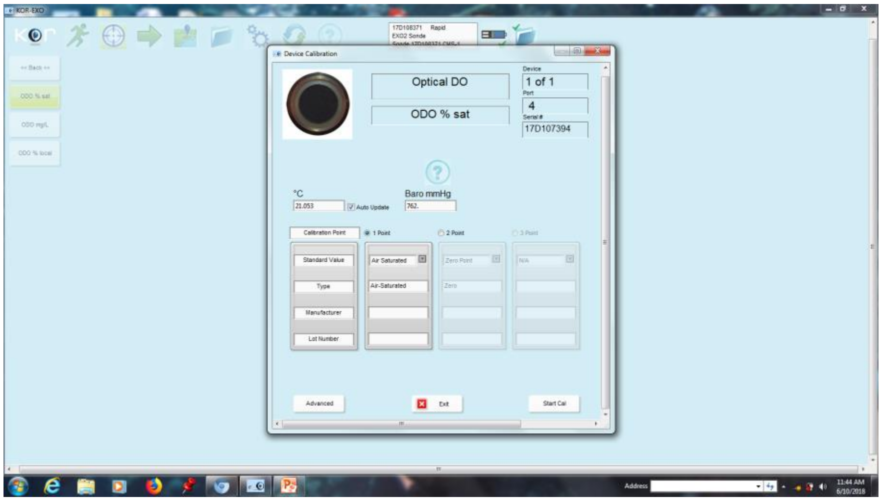

Figure 6-2. Using air-saturation for Dissolved oxygen calibration.

Fill the cal cup with ½ in of water, and place the sonde in the cup, but do not screw the collar on. The pressure in the cup must be atmospheric pressure so the cup must be open to the atmosphere.

To begin calibration, click the “cal” icon, then ODO (Optical Dissolve Oxygen), then hit “% saturation”. Fill in the form, including the atmospheric pressure as obtained above and click start. When the readings stabilize (Figure 6-3), hit complete. Look for green check, view and print worksheet (Figure 6-4).

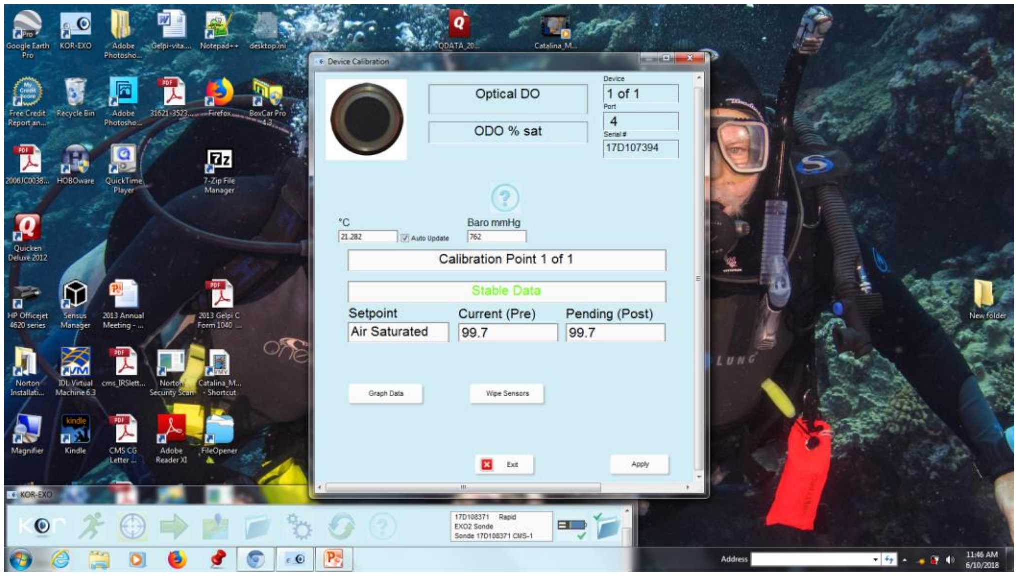

Figure 6-3. ODO cal stabilization.

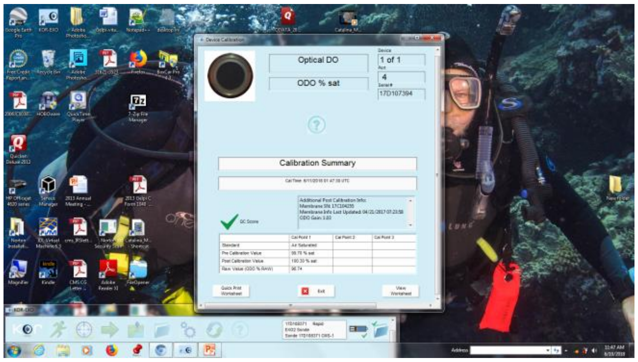

Figure 6-4. View and print worksheet.

7. Depth setting

We typically will not calibrate the depth sensor.

8. Chlorophyll Calibration

We typically will not calibrate the chlorophyll sensor.

9. Recording Calibration Results

Every time the instrument undergoes a calibration, the calibration results, that is the worksheets, must be placed on the CMS website. They can be printed and scanned, or viewed and screen dumped with the figures or pdf emailed to the CMS webmaster.
We want a history of the calibration. It is reviewed and helps us understand the health of the sensors, when they should be replaced, how fast do they lose calibration and much else. Even if the deployment is cancelled, the calibration data should be placed on the website, which is our official record.

10. Deployment Settings

Usually, the sonde's sensors will be calibrated in preparation for a deployment, although they may also be tested after returning from a deployment if the data appear unusual and we want to verify the sensors were working correctly. In the former case, after recording the calibration results, the sonde will be programmed for the next deployment.

The deployment procedure is to:

1. upload a data file from the PC to the sonde, indicating what parameters are to be collected (logged) when and at what sampling rate;

2. set the time to begin data logging.

To begin the deployment programming, click the green arrow at the top of the KOR application. Three options will appear, including "Open a template" (see Figure 10-1). A list of files will appear from the KOR directory on the PC. Select the one corresponding to the type of deployment to be made. If none fits the deployment, choose one that is close and you can modify it and save it later. Hit load and the file will be transferred to the sonde.

The settings will now appear in the window. They can be changed by clicking the tool icon above the settings window (Figure 10-2). Typically, the site name will change, but there may be other changes as well.

Check the sonde settings by clicking "Read current sonde settings" (Figure 10-3).

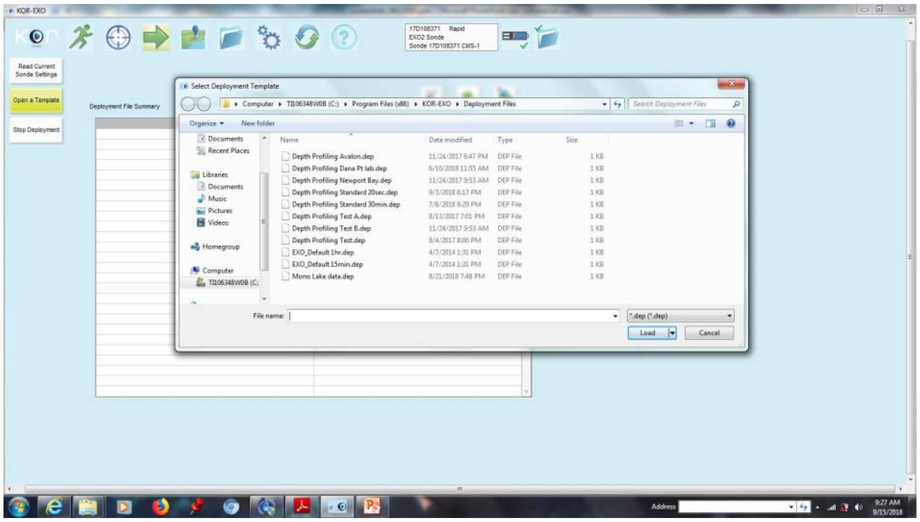

Figure 10-1.

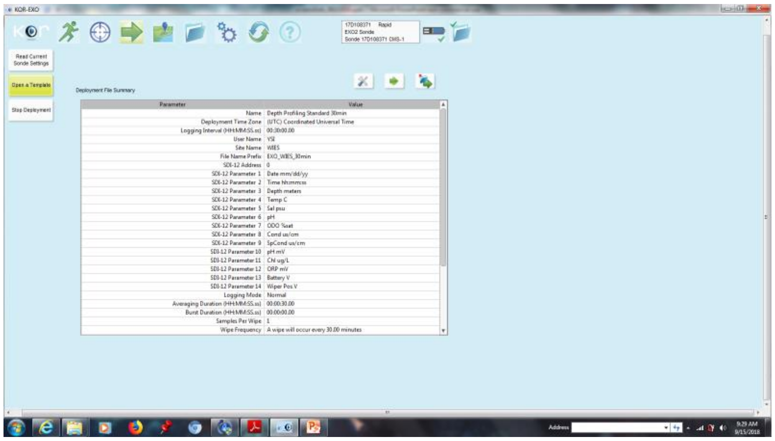

Figure 10-2. Data to be recorded.

Note the logging time settings.

You can click the green arrow above the settings (but not the one at the top of the page) to set the logging times.

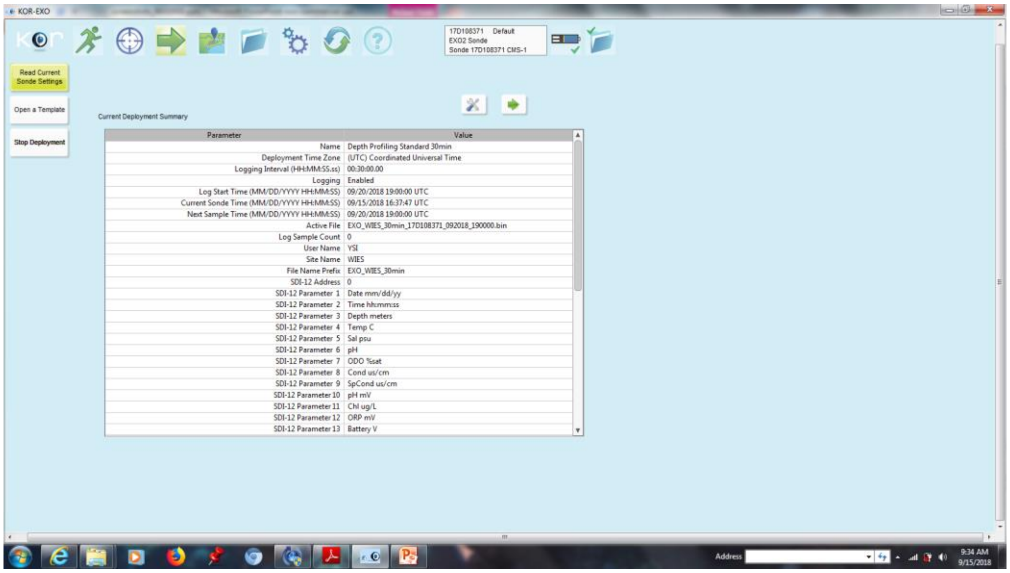

Figure 10-3. Sonde settings.

When setting the start logging time, note that there are 3 choices, Figure 10-4. Typically, we will choose the “Custom Time”. Fill in the hour and date. Note that we use UTC time. You will have to determine the local time you wish to begin logging, then usually add 7 or 8 hours to that time to get UTC time. You can find the difference by noting the sonde time “Now” and your computer’s time.

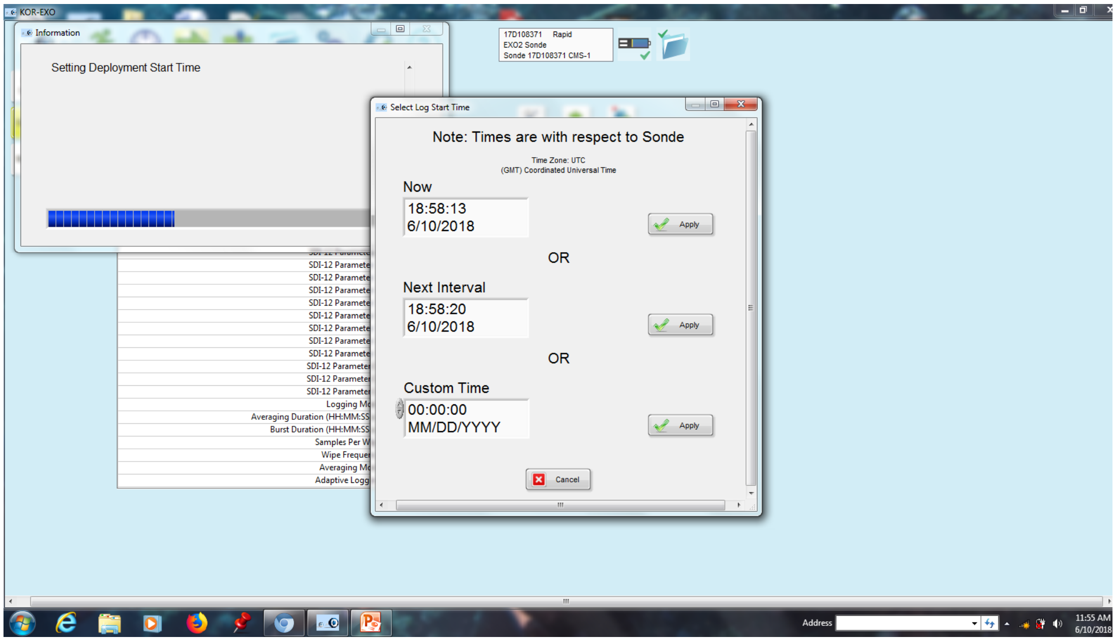

Figure 10-4. Choosing the start time

Once the time is set, hit “Apply”. The sonde is now on deployment, though it will not log data until the Custom Time arrives. You can now cancel out of the KOR application.

11. Maintenance TBD
