# Supplementary material for: Dynamics of pH at Santa Catalina Island
Source: PLoS One. 2023 Dec 7;18(12):e0290039. doi: 10.1371/journal.pone.0290039 (PMC10703214; doi:10.1371/journal.pone.0290039)
Supplement: Attachment — Submitted filename: Comments on Dynamics of pH at Santa Catalina Island.docx [file pone.0290039.s039.docx]

Comments on "Dynamics of pH at Santa Catalina Island"

This manuscript studies the pH of the fixed-depth and depth-profile at Santa Catalina Island and analyzes its dynamics. The author found that the pH value is higher than that obtained from reports of pH at coastal locations but similar to open-ocean values reported for the bight. And the author concluded that the pH depth gradient increased with temperature stratification and that the major temporal variation in pH was associated with internal waves. 
As a whole, the manuscript is well arranged. The results showed pH at Santa Catalina Island, which is situated within the Southern California Bight. And the conclusions may be helpful for understanding how pH can vary at specific sites due to oceanographic processes. But the results and discussion are not adequate. There are still some defects and questions in this manuscript. Therefore, I suggest accepting this manuscript if the following questions or errors are well fixed by the author:

(1) Most of the content of the manuscript only displays the observation results, which is more like a note or report than a research article. The manuscript requires some statistical analysis to explain the results. For example, if you want to demonstrate the correlation between temperature, salinity, and pH, you should provide the correlation coefficient between them (Lines 175-177, Lines 188-190, Lines 290-291, etc.). There is too much information in figures 9 and 10, and it is hard to visualize how they relate to each other. It is better to use another figure or table to show the relationship between temperature and pH profiles ("larger pH depth gradients correspond with greater temperature stratification," the sentence in Lines 287-288).
(2) Some abbreviations did not show their full name when they first appeared, i.e., "SCUBA" in the Abstract, "YSI" in Line 78, "SST" in Line 101, "CDOC" in Line 102,... 
(3) There are quite a few sentences that need support from a figure or reference (Lines 174-177, Line 219, etc.); therefore, discussion needs to be further enhanced. In addition, corresponding evidence shall be provided for upwelling and downwelling. 
(4) Lines 66-71, literature review needs to be more specific and targeted. 
(5) Lines 165-167, To say "strong summer stratification conditions" and "well-mixed winter conditions", it is better to give readers the mixed layer depth in summer and winter.
(6) Figure 1, Why choose SST? Land should be marked with other colors. The location of the mooring chain and sampling points should be labeled on the map. 
(7) Lines 174-177, where did you get that? 
(8) It seems that you did not show the relationship between chlorophyll, dissolved oxygen and pH. How did you determine the impact of biological activity on pH?

(9) Lines 230-232, Is the content mentioned above (Lines 160-226) just the extremes? If so, then the conclusion does not have dependability. 
(10) Figure 7, what is the point of this figure? 
(11) Figures 9 and 10 can be combined together.
