# Supplementary material for: Dynamics of pH at Santa Catalina Island
Source: PLoS One. 2023 Dec 7;18(12):e0290039. doi: 10.1371/journal.pone.0290039 (PMC10703214; doi:10.1371/journal.pone.0290039)
Supplement: Attachment — Submitted filename: Response to Reviewers.docx [file pone.0290039.s040.docx]

Response to Reviewers regarding PONE-D-23-12055

“Dynamics of pH at Santa Catalina Island.”

Below are the reviewers’ comments and my description of changes to the paper in response to them (in blue text).

Reviewer #1 comments and responses

1. pH may be relative to the temperature, conductivity, chlorophyll and dissolved oxygen. Would you give the relation between pH and these parameters? That is to say, multivariate statistical analysis should be added.

I have added 2 graphs showing the computation of coherence as a function of frequency between temperature, pH, dissolved oxygen and chlorophyll. Analysis of these graphs does not change any conclusions in the paper.

2. Internal waves and upwelling should be elaborated in the Introduction.

I have elaborated on the phenomena of general upwelling and upwelling produced by internal waves in the Introduction lines 57-61.

3. Results and discussion should be divided into the independent Results and discussion.

The Results and Discussion were separated in the original manuscript (see line 407). However, the Discussion heading appeared at the bottom of the page. Its location is now changed (line 408) to better signify its position within the text. See lines:

4. What do the Internal waves and upwelling play an important role on pH?

This is discussed in the Discussion section at length. Lines: 466-475 and 481-487..

**Reviewer #2 comments and my respones**

1. Most of the content of the manuscript only displays the observation results, which is more like a note or report than a research article. The manuscript requires some statistical analysis to explain the results. For example, if you want to demonstrate the correlation between temperature, salinity, and pH, you should provide the correlation coefficient between them (Lines 175-177, Lines 188-190, Lines 290-291, etc.). There is too much information in figures 9 and 10, and it is hard to visualize how they relate to each other. It is better to use another figure or table to show the relationship between temperature and pH profiles ("larger pH depth gradients correspond with greater temperature stratification," the sentence in Lines 287-288).

Additional statistical analyses in the form of spectral coherences are now included, see new figures 4 and 5. Additionally, the correlation coefficients between variables is now computed and given in the panels of the new figures. Figures have been combined to be the new Figure 11, that should make a comparison between pH gradients and temperature gradients easier.

2. Some abbreviations did not show their full name when they first appeared, i.e., "SCUBA" in the Abstract, "YSI" in Line 78, "SST" in Line 101, "CDOC" in Line 102.

SCUBA is now defined in the abstract, line 24. YSI is the company’s name, not an abbreviation. CDOC was defined in the original manuscript, line 47, the first time it was used, and remains so in the revised manuscript. SST is now defined line 98. Also, dissolve oxygen (DO) and chlorophyll (Chl) are now used and defined.

3. There are quite a few sentences that need support from a figure or reference (Lines 174-177, Line 219, etc.); therefore, discussion needs to be further enhanced. In addition, corresponding evidence shall be provided for upwelling and downwelling.

A direct reference is made to Fig 2 regarding correlations obtained from inspection. In addition, two figures describing coherence as a function of frequency have been added (Figures 4 and 5) with some discussion to further indicate the correlation. Additionally, the correlation coefficients of the entire time series are also included as text within the figures 4-5. Also, the fact that increasing temperatures correspond to downwelling and decreasing temperatures correspond to upwelling, as evidenced by the time series plots is made explicit, lines 171-172.

4. Lines 66-71, literature review needs to be more specific and targeted.

This section has been rewritten to be more explicit regarding the references and their relation to the paper. See lines 52-55 and lines 67-71.

5. Lines 165-167, To say "strong summer stratification conditions" and "well-mixed winter conditions", it is better to give readers the mixed layer depth in summer and winter.

Estimates of the mixed-layer depth are now included, lines 166 and 240-241.

6. Figure 1, Why choose SST? Land should be marked with other colors. The location of the mooring chain and sampling points should be labeled on the map.

SST is chosen to supplement the discussion, as it can identify upwelling areas. This is now explicitly stated in the text when the figure is introduced. The mooring chain and heavily sampled areas are marked with T and A, respectively. Additionally, we have placed a white dot on the locations.

7. Lines 174-177, where did you get that?

The correlations between pH, temperature and salinity are obvious from figure 2. However, to make it more explicit, the coherence calculations are now shown in the new figures 4 and 5.

8. It seems that you did not show the relationship between chlorophyll, dissolved oxygen and pH. How did you determine the impact of biological activity on pH?

The relationship between dissolve oxygen and pH, as well as chlorophyll and pH are now shown via coherence in figure 5. The coherences (and correlation coefficients) are low. This is now mentioned in the text.

9. Lines 230-232, Is the content mentioned above (Lines 160-226) just the extremes? If so, then the conclusion does not have dependability.

I do not understand the reviewer’s comment. If the same phenomenon produces the extremes, we would expect it to produce the results between the extremes. This is also consistent with the original figure 12.

10. Figure 7, what is the point of this figure?

This figure is now numbered 9. A depth profile is obtained from single-depth measurements, a somewhat novel method. We feel these results should be isolated and be available for reader scrutiny. The figure also indicates the depth of the mixed layer as determined by measurements at a single depth. This is now explicitly indicated in the new text.

11. Figures 9 and 10 can be combined together.

The combined old figures 9 and 10 are now the new Figure 11.
